# Supplementary material for: Prognostic Genomic Tissue-Based Biomarkers in the Treatment of Localized Prostate Cancer
Source: J Pers Med. 2022 Jan 7;12(1):65. doi: 10.3390/jpm12010065 (PMC8781984; doi:10.3390/jpm12010065)
Supplement: Supplementary file 1 [file jpm-12-00065-s001.zip › jpm-1474373-supplementary.pdf]

Table S1 (Supplemental material). Newcastle Ottawa Scale for risk of bias assessment of the included studies (scores ≥7–9, 4–6, <4 are considered as low, intermediate, and high risk, respectively)

Study	Selection				comparability	Outcome			overall
	Rappresentativeness of exposed cohort	Selection of non-exposed	Ascertainment of exposure	Outcome not present at start		Assessment of outcome	Adequate follow-up lenght	Adequacy of follow-up	
Erho 2013 [12]	*		*			*	*	**	6/9
Karnes 2013 [13]	*		*			*	*	**	6/9
Cooperberg 2014 [14]	*		*			*	*	**	6/9
Ross 2014 [15]	*		*			*	*	**	6/9
Den 2014 [16]	*		*			*	*	**	6/9
Klein 2015 [17]	*		*			*	*	**	6/9
Ross 2016 [18]	*		*			*	*	**	6/9
Den 2015 [19]	*		*			*	*	**	6/9
Cuzick 2011 [23]	*		*			*	*	**	6/9
Bishoff 2014 [24]	*		*			*	*	**	6/9
Freedland 2013 [25]	*		*			*	*	**	6/9
Cuzick 2015 [27]	*		*			*	*	**	6/9
Cooperberg 2013 [26]	*		*			*	*	**	6/9
Canter 2020 [28]	*		*			*	*	**	6/9
Klein 2014 [34]	*		*			*	*	**	6/9
Van Den Eeden 2018 [36]	*		*			*	*	**	6/9
Brooks 2021 [37]	*		*			*	*	**	6/9
Covas Moschovas 2021 [38]	*		*			*	*	**	6/9
Cullen 2021 [35]	*		*			*	*	**	6/9
